# The Relevance of Internal Working Models of Self and Others for Equine-Assisted Psychodynamic Psychotherapy

**DOI:** 10.3390/ijerph191710803

**Published:** 2022-08-30

**Authors:** Géza Kovács, Annemiek van Dijke, Roeslan Leontjevas, Marie-José Enders-Slegers

**Affiliations:** 1 Department of Psychology, Open University of the Netherlands, 6419 AT Heerlen, The Netherlands; 2 SPEL Psychologen Putten, 3881 NE Putten, The Netherlands; 3 PsyQ I-Psy Brijder The Netherlands, Leiden University Medical Centre, 2333 ZA Leiden, The Netherlands

**Keywords:** equine-assisted psychotherapy, psychodynamic, attachment, trauma, internal working models

## Abstract

Attachment characteristics play a key role in mental health and in understanding mental disorders. The aim of this study was to gain insight into the role the attachment characteristics can play in treatment effects in adult patients with intrapsychic and interpersonal problems who underwent Equine-assisted Short-term Psychodynamic Psychotherapy (ESTPP). In the first part of the study, we compared the effects of ESTPP to treatment-as-usual from a previous dataset regarding psychological dysfunction. For this, an explorative experimental non-randomized pre-treatment and 1-year post-treatment design was used. A mixed model revealed a significant decline in psychological dysfunction for both conditions, with no significant difference between the two. In the second part of the study, we examined the course of ESTPP effects over the period of 1 year when controlled for attachment styles and, subsequently, for internal working models of self and others. To this end, measurements were taken at baseline, 2 months waiting time, one-week intensive module, 6 months, and one year after the start of the treatment. Mixed models accounted for repeated measures showed significant improvements in psychological dysfunction, remoralization, and depression for ESTPP patients over time. The study implies that models of self and others may be used to predict the course of effects, which is relevant in determining what works for whom. In particularly, duration and intensity of therapy and a focus on the Model of Self seem relevant for shaping a more personalized treatment. ESTPP seems beneficial for patients with low pre-treatment attachment security.

## 1. Introduction

The efficacy of psychotherapy for all kinds of mental disorders—including the aftermath of traumatization and personality problems—has improved over the years [1,2,3,4,5,6,7]. The integration and mutual underpinning of attachment theory, psychotherapy, and neurobiology show that attachment plays a central role in mental health. Secure attachment serves as protection against life’s adversities, and shapes the accessibility of the autobiographical memory, the capacity for coherent thinking or problem-solving, the capacity to see new experiences and thoughts in a new light, namely the capacity for metacognition and mentalization. In insecure attachment, these seem impaired [8,9,10,11,12,13]. Insecure attachment experiences, when intertwined with other adversities in the developing years of the person(ality), are associated with decreased resilience to life’s difficulties and a range of mental disorders later in life [14,15,16,17].

A meta-analysis by Levy et al. [18] suggests that patients with secure attachment pre-psychotherapy show better psychotherapy outcomes than insecurely attached patients. Furthermore, an understanding of the intertwinement of trauma, personality problems, and unsafe attachment experiences associated with Complex Post-Traumatic Stress Disorder (CPTSD) and/or Disorders of Extreme Stress Not Otherwise Specified (DESNOS) and/or Developmental Trauma Disorder (DTD) has shaped the focus of psychotherapy in general [16,19,20,21,22,23,24,25,26,27,28].

The concept of attachment refers to the early interaction experiences of an individual with significant others (parents or primary caregivers) [16,29,30,31], which create an image of the self and others and support systematic patterns or “internal working models” of interpersonal relationships, emotions, and behavior. These internal working models are based upon an internalization of a history of autobiographical interpersonal experiences with significant others, such as parents and primary caregivers [16,29,30,31]. These models may be applied in the context of various theoretical frameworks and clinical approaches that incorporate experiential forms of interventions. These experiential forms seem to be relevant for therapy of complex trauma in the context of the therapeutic relationship [23,27].

Bartholomew and Horowitz [32] describe attachment styles as the results of two-dimensional concepts of internal working models—namely, the Model of Self (MoS) and the Model of Others (MoO)—that facilitate a four-category model of attachment styles (behavior). With a positive internal working model of oneself (positive MoS), a person considers themselves worthy of being loved and supported by others in times of distress and capable of facing difficulties. However, this self-esteem is lacking where there is a negative working model of oneself (negative MoS). With a positive MoO, a person sees others as reliable and available to call on in times of distress and when facing difficulties. With a negative MoO, a person views others as unreliable and dismissive and as malfunctioning in facing one’s difficulties in life. A combination of these working models produces four dimensional categories of attachment: *secure* (positive MoS, positive MoO), *preoccupied* (negative MoS, positive MoO), *dismissive* (positive MoS, negative MoO), and *fearful* (negative MoS, negative MoO) [32]. (The fearful attachment style is also known as disorganized attachment style).

To overcome psychological problems, psychotherapy seeks to create an intersubjective therapeutic relationship in which affect-regulation can take place in the presence of a reflective other. The psychotherapeutic relationship has similarities with a secure parent–child attachment [11,23,33,34].

However, an established and distorted Model of Self and Others (MoS and MoO) in patients with attachment-related problems such as CPTSS, DESNOS, or DTD makes it difficult to establish long-term, productive, intersubjective relationships—in effect, to engage in the therapeutic healing process [16,20,35]. Many patients in this population jump from one therapy to another, reflecting a sense that the treatments are not in line with their needs [36,37,38,39]. Amongst others, the field of Animal-Assisted Psychotherapy (AAP) seeks ways of facilitating the psychotherapy process by the inclusion of animals in the therapeutic process [35,37,38,39,40,41,42,43].

### 1.1. Animal-Assisted and Equine-Assisted Psychotherapy

AAP was first introduced by the psychologist Levinson and has been gaining recognition in the clinical world [44,45]. AAP may be carried out in the context of various theoretical frameworks integrating animals into their setting. Various kind of pets or domesticated animals are included. The integration of the animal brings up projections and emotions through its authenticity and aliveness [35,45,46].

AAP is a clinical field based on accepted principles and goals of evidence-based psychotherapy and incorporating various theoretical frameworks with Equine-Assisted Psychotherapy (EAP) as a subcategory [35,47,48]. Here, a therapeutic triad is formed in which the horse supports the therapeutic relationship and techniques to benefit the patients’ intra- and inter-psychological processes. EAP is an experiential form of therapy in which activities with the horse—alongside as well as on the horse—are executed and reflected on by a therapist. The horse can be introduced into the therapy as a non-verbal reciprocal transference and transitional object in order to create corrective emotional experiences for the patient [40,41,49]. The field of AAP in general [35,45,50,51,52] and of EAP in particular [37,41,49,51,53,54,55,56,57,58] have depicted the benefits of the inclusion of the horse in therapy. Animal-assisted psychotherapy could provide for sensory experiences due to physical interactions (petting, brushing, etc.) between patient and animal through which neurodevelopmental areas can be reached [35,59]. Several specific features of horses make them suitable for therapeutic interventions, especially in the psychodynamic domain. First, their size and strength which can be intimidating and provoke certain behavior and projections in and by patients. Due to their size and strength, it is possible to be carried, resembling the rocking experience between parent and child. Due to the bilateral, rhythmic, patterned repetitive movement, in horseback riding, patients could become passively regulated, organizing the lower regions of the brain [58]. An experience which many patients with unsafe attachment experiences have missed in their developmental years. Further, horses are social animals living together in a herd with each horse having its own personality and behavior, resembling human social life [58]. Horses are prey animals, possibly resembling patients with unsafe attachment experiences who could have felt (still feel) as prey themselves [43]. When patients are invited to work with animals in therapy, patients are helped to be in the hereandnow, which provides for calm states in the limbic system necessary to foster left-right brain connections, connecting causal explanations to emotional and sensory experiences [58]. Lastly, horses (as well as dogs) are considered to be capable in recognizing human facial expressions, providing for an extra characteristic to form an interspecies relation [60]. In contrast to dogs, horses will not express an unrelenting enthusiasm and affection as dogs do [61,62]. Horses show more autonomy (whereas dogs appear to be more symbiotic) [54,62], which may appeal to the mentalization skills of the patient when engaging with a horse.

Moreover, in conjunction with the biophilia hypothesis by Wilson [63] stating that humans have an innate need for deep and intimate association with animals, Mormann’s study [51] implies that patients may be more naturally disposed to be in touch with their inner world in the presence of an animal. Thus, the horse in EAP might serve as an enabler into the client’s emotional world [35,51,60]. Therefore, and due to the potential of interspecies relationships between human and animal [64,65], the creation of an intersubjective relationship with the horse and the therapist as reflective of others seems possible [50]. Hence, the added value of EAP could be the addition of human–animal interactions in addition to human–human interactions (patient–therapist). It might be that through the human–animal interactions the attachment characteristics, i.e., MoS/MoO, can be magnified for the patient, as it were a magnifying glass. This magnification arises from the projections and reflections by the patient in the interaction with the horse which mirrors aspects of how the patient views himself and others, suitable for more psychodynamic oriented EAP.

Through these horse–human interactions—which can be perceived as non-judgmental and not hindered by (human–human) language—the patient’s experiences and interpretation might be more easily related with and by the clinician to the internal models of self and others. In other words, this nonverbal interaction can help to amplify the patient’s poor mentalizing capacity regarding self-other-insight and understanding. The explicit features of the horse may therefore elicit attachment and mentalization themes on cognitive-emotional and bodily-emotional levels, as well as influencing muscle-motor activity in patients, as seen in “parent–child interactions” [50,55,57].

Patients with secure attachment styles are generally able to deal actively and constructively with negative affect and take advantage of the enhanced creativity made possible by positive affect. Although the therapeutic process is challenging for anyone engaging in psychotherapy, these patients remain largely within the “window of tolerance” [13,16,22]. They find new and unusual ways of dealing with challenging events, enjoying the task performance and maintaining a positive mood. In contrast, insecure attachment-based de-activating strategies seem to distance people from their own emotions, averting the painful experience of the negative affect but also foregoing the benefits of a positive affect [27]. Insecure attachment-based hyper-activating strategies seem to generate an arousal level that overwhelms the patient with emotions, without providing the accompanying mentalization capacities [16,25,30,66]. The presence of and interaction with animals may calm the neurological system associated with the insecure attachment style, allowing the patient to experience and express their emotions and engage in self-regulation, while also enabling more adequate coping strategies [35]. Subsequently, there can be a process of learning to stay within the window of tolerance while experiencing self-agency and -mastery.

Patients with a preoccupied attachment style, thus *negative MoS with positive MoO* (i.e., insecure attachment-based hyper-activating strategies, characterized by a high affective mental arousal and accompanied by poor mentalization skills) need validation of their emotions and initiation of their separation and individuation [16,67]. In EAP, the horse can function as an accepting and responsive Other (alongside the therapist), due to the horse’s features and capability for relating to the emotional level [35,57,68]. This may enable the validation of the patient’s emotions and strengthening of their trust in themselves.

Patients with a fearful attachment style, thus *negative MoS with negative MoO* (i.e., a combination of insecure attachment-based hyper-activating and de-activating strategies, characterized by difficulty trusting other people and tolerating closeness) need careful attunement [16,67]. In EAP, the horse may serve as a safe reciprocal practice opportunity for the patient to explore closeness [35,57].

Patients with a dismissive attachment style, thus *positive MoS with negative MoO* (i.e., insecure attachment-based de-activating strategies, characterized by socio-emotional detachment) need help in understanding the relationship between (inter)personal stress, body sensations, and affect [16,67]. In EAP, the horse may facilitate experiential, non-verbal interactions and socio-emotional processing [49,57].

A study by Kovács et al. [41] showed that an integrated Equine-assisted Short Term Psychodynamic Psychotherapy (ESTPP) (short term indicates a limited duration of therapy with an emphasis on the termination phase of therapy [69]) is feasible and effective for a patient group with personality problems seeking to overcome interpersonal sensitivity and enhance self-efficacy and self-esteem, in other words, strengthen MoS. However, despite the above, a systematic review by Kovács et al. [70] revealed that the EAP field remains in its infancy, particularly in relation to research on attachment-related mental problems in adult patients.

### 1.2. Study Aims

There is a general agreement among clinicians, researchers, and patients on the need for more personalized psychotherapy options in order to know “what works for whom” [71,72]. In this vein, several studies have examined how attachment representations or styles may relate to psychotherapy outcomes [33,73,74,75]. However, no studies have examined the use of Equine-Assisted Psychotherapy with adult patients. In addition, because of the patient’s projections and reflections in the interaction with the horse, which could reflect aspects of how the patient sees himself and others, ESTPP could clearly differentiate internal working models MoS/MoO. In accordance with the Parish–Plass [35] findings on the theoretical benefits of AAP for patients suffering from developmental trauma—and to extend the research on EAP—the aim of this naturalistic study was to contribute to the knowledge concerning *which patient benefits the most from ESTPP depending on the patient’s attachment characteristics.*

The first part of this study aimed to compare the effects of ESTPP and treatment-as-usual (TAU) on patient outcomes in psychological dysfunction. In the second part, we examined the process of ESTPP by differentiating for insecure attachment styles and subsequently differentiating for internal working models (MoS/MoO). Such insights could help with identifying options for personalized psychotherapy. Securely attached patients are assumed to have better psychotherapy outcomes than insecurely attached patients [73], therefore this study contributes to the literature by differentiating for insecure attachment styles (i.e., fearful, dismissive, and preoccupied) and secure attachment style and their respective patient journeys along the internal working models (MoS/MoO) in ESTPP. Since depression is related to negative MoS [17,76,77], the effects on depression and remoralization were estimated alongside those on psychological dysfunction for the patients undergoing ESTPP. Depression can be seen as a state of mind that inhibits the zest for life, whereas remoralization is the opposite state of mind, in which there is hope for and confidence in the future [78].

## 2. Method

### 2.1. Design

The first part of the study examined the effects of ESTPP using an explorative experimental non-randomized pre- and post-treatment design, with two intervention groups (ESTPP versus TAU). In both conditions, the therapy was terminated around (but not later than) 12 months after the start.

The second part of the study examined the course of therapeutic effects of ESTPP during the year. For the ESTPP condition only, the outcome variables psychological dysfunction, remoralization, and depression were examined. The effects were controlled for attachment styles, MoS and MoO. Measurements were taken at 2 months prior to the start of the therapy, before the start of the therapy (baseline), after 1 week of the intensive therapy module, at 6 months after the start of the therapy, and at 12 months after the start. Table 1 shows the designated time points of the two study parts with the instruments used.

### 2.2. Participants and Procedure

#### Inclusion and Exclusion Criteria

The participants were recruited from a group who were to undergo ESTPP at the mental health care center Ars Curae/SPEL Psychologen Putten (AC), a psychotherapy facility designed to offer the ESTPP program, as well as from another group who were to undergo TAU at Zaans Medisch Centrum (ZMC), a mental health center. The AC and ZMC offices are located in the same region, share a psychodynamic psychotherapy view, have back and forth patient referrals, and could be considered sister organizations. All potential participants were referred by their physician (in mutual consultation) to either AC or ZMC for the specialized psychotherapy. During the intake procedure at both facilities, the diagnoses and therapy indications were established. In general, patients referred to these facilities have a variety of psychological complaints, characterized by a high severity and complexity of intrapsychic and interpersonal problems, as classified by the Diagnostic and Statistical Manual of Mental Disorders (DSM-5) [79].

During the intake procedure, the participants were informed about the psychotherapy conditions and provided written informed consent to participate in the study prior to inclusion, in line with the Declaration of Helsinki. Participants had to be 18 years or older, and those in the experimental condition had to be able to travel independently abroad. All patients included in the study had attachment-related personality problems. The exclusion criterion was inadequate psychological stability indicated by suicidal behavior, psychosis, or substance abuse.

For the ESTPP condition, 193 participants were enrolled with a score on attachment style and at least one score of the outcome variables. Of these, *N* = 161 (83.4%) were female, and the average age was 37.6 years (SD = 12.4), ranging from 17 to 81 years. There were 107 participants in the TAU condition, *N* = 78 (72.9%) were female, and the average age was 30.5 years (SD = 10.9), ranging from 17 to 58 years. Data from the participants of TAU were obtained from the SPECTRE study, an extensive multi-centered study on psychotherapy effectivity in patients with personality disorders [80,81]. The study was approved by the Medical Ethics Committee of the VU University Amsterdam Medical Center in the Netherlands.

### 2.3. Intervention

The ESTPP was delivered in a 1-week (6-days) intensive individual (inpatient) module at a remotely located ranch in Spain/France, followed by outpatient (ambulant) weekly sessions of 1 h, at descending frequency (assessed by the treatment team), up to the end of the trajectory (12 months, around 25 sessions) in the Netherlands at which the acquired insights were further processed. The 1-week intensive module consisted of four 2-h daily sessions, and the ambulant sessions were 1 h in length. The trajectory consisted of a diagnostic or stabilization phase, a focal phase, a consolidation phase, and termination phase and it had an experiential character in which the therapists, who are additionally trained by animal experts, reflected on what was occurring between the patient, the horse, and the therapist [57]. The sessions were well-structured, with a balance between therapeutic exercises and rest, with set eating and feeding times for, respectively, patient and animals. Exercises with the horses—alongside as well as on the horse—consist of guided tasks in observation, (physical) contact, tuning into the affective state of the horse, finding synchronicity and dealing with the instantaneous feedback of the horse, leadership, congruence, body posture, ‘letting go’, relaxation, concentration, setting boundaries, dealing with fear and longing, balance and taking control, and taking care of oneself and the animal. These experiences in the patient’s here-and-now are metaphorically related to patient’s daily life and core-conflict with the help of the therapist [44,58].

The control condition (TAU) was a multi-modal inpatient group therapy including individual psychotherapy Short Term Psychodynamic Psychotherapy [STPP]) and STPP-informed modules of expressive therapies, such as art-therapy, psychodrama, and psychomotor therapy. The therapy took place over 9–12 months, for 3 days per week.

### 2.4. Measurements

Psychological dysfunction was measured using the Dutch version of the *Brief Symptom Inventory* (BSI) [82,83] a validated self-report scale derived from the revised Symptom Checklist-90 [84,85]. The BSI is a self-report questionnaire consisting of 53 items and a 5-point Likert scale, ranging from 0 (not at all) to 4 (extremely). Rankings characterize the intensity of distress during the past seven days.

The total score on the psychological dysfunction scale was used in this study. A higher norm score on the range of 1–7 indicates more severe dysfunction. The BSI is a useful tool for measuring progress during and after psychotherapy and has been shown to have good validity and reliability, with a Cronbach’s alpha of 0.96 [83]. The alpha value in our sample was 0.82. The BSI was used in study part 1 and 2.

Remoralization was measured using the *Remoralization Questionnaire* (*RQ*). The RQ is a self-report inventory and was used to measure hope and confidence or trust. The questionnaire contains 12 questions and a four-point scale (1 = completely disagree to 4 = completely agree). The higher the score on the RQ, the more hope and confidence or trust the participant has. The RQ has a high Cronbach’s alpha of 0.91 and good reliability (r = 0.89) [78]. The alpha value in our sample was 0.88. RQ was used in study part 2.

The severity of depressive symptoms was measured using the Beck depression inventory (BDI). The BDI is a self-report inventory consisting of 21 items, each item rated by the patient on a four-point scale, ranging from 0 (symptom not present) to 3 (symptom very intense). For example, (0) “I don’t feel disappointed in myself,” (1) “I am disappointed in myself,” (2) “I am disgusted with myself,” and (3) “I hate myself.” A total score of 0–13 indicates that a person is not depressed, 14–19 indicates mild-moderate depression, 20–28 indicates moderate-severe depression, and 29–63 indicates severe depression. The BDI has demonstrated good validity, with high values for Cronbach’s alpha (0.86) and for psychiatric and nonpsychiatric populations (0.81) [86]. The alpha value in our sample was 0.97. BDI was used in study part 2. 

Adult attachment styles were measured using the attachment style questionnaire (ASQ, Dutch HVL) [87,88] in the ESTPP condition. This questionnaire includes 38 items and a five-point scale (1 = completely disagree, 5 = completely agree). The subscales represent the four attachment styles: secure, fearful, dismissive, and preoccupied. A score of between 1 and 5 is calculated for each of the scales, and the participant’s highest score determines his or her predominant attachment style. The validity of the tool is reasonable, and reliability is reasonable-to-good (HVL) [87,88]. Previous research has shown a Cronbach’s alpha of 0.75 for the secure attachment style, 0.80 for preoccupied, 0.79 for fearful, and 0.62 for dismissive [89]. In our sample, alpha levels were for the secure style 0.88, for the preoccupied style 0.92, for the fearful style 0.90, and for the dismissive style 0.91. ASQ was used in study part 2.

Based on the four attachment style categories, the dummy variables for MoS and MoO were computed, following Griffin and Bartholomew [90]. In Table 2, the attachment styles with a positive MoS were coded with 1 (i.e., secure and dismissive share a positive MoS), and attachment styles with a negative MoS (i.e., fearful and preoccupied) were coded with 0. Subsequently, the attachment styles with a positive MoO (i.e., secure and preoccupied) were coded with 1, and attachment styles with negative MoO (i.e., fearful and dismissive) with 0.

### 2.5. Statistical Analyses

IBM SPSS Statistics for Windows, Version 25 was used IBM Corporation, Armonk, USA (IBM Corporation, Armonk, NY, USA). Descriptive analyses in SPSS were run to assess the baseline characteristics of the study population. 

To assess the difference in effects between ESTPP and TAU on psychological dysfunction (study part 1), we built a mixed model that accounted for repeated measures within the participants with a psychotherapy condition (ESTPP compared to TAU) as predictor and time-point T4 compared to time point T1 (as a categorical variable, fixed effect). 

For the ESTPP condition (study part 2), we, first, built models with time-points for psychological dysfunction, remoralization, and depression. For interpretation of the effects at 12 months compared to the effects at one week after the intensive treatment module, the analyses were re-run with T2 as reference. 

Furthermore, we built models with: (1) the four attachment styles, (2) MoS, and (3) MoO. As in part 1, linear mixed models accounted for repeated measurements. These models were extended with the interaction terms between time on the one hand, and the attachment styles with secure attachment as reference, MoS and MoO with positive models as reference on the other hand. We adjusted all models for age and sex. For all models, patients with a non-missing attachment style and a score on at least one of the outcome variables were included in the analysis. Due to missing scores, measurements differed in N for specific outcome variables. Missing scores were not imputed.

The two-tailed significance level was set at α = 0.05. To understand the magnitude of the differences found, we also reflected on the size of the estimated effects. Standardized effect sizes (*d*) were calculated by dividing the estimated effect of the intervention by the standard deviation at baseline. Here, a *d* of <0.20, 0.20–0.49, 0.50–0.79, and >0.80 were interpreted as negligible, small, moderate, and large, respectively [91].

## 3. Results

A descriptive of the study populations is presented. Table 3 shows the baseline scores on psychological dysfunction for ESTPP and TAU, and subsequently baseline scores on remoralization, depression, and the distribution of attachment styles in the ESTPP (*N* = 193) condition. 

### 3.1. Difference in Effects of ESTPP and TAU on Psychological Dysfunction

**Figure 1:**
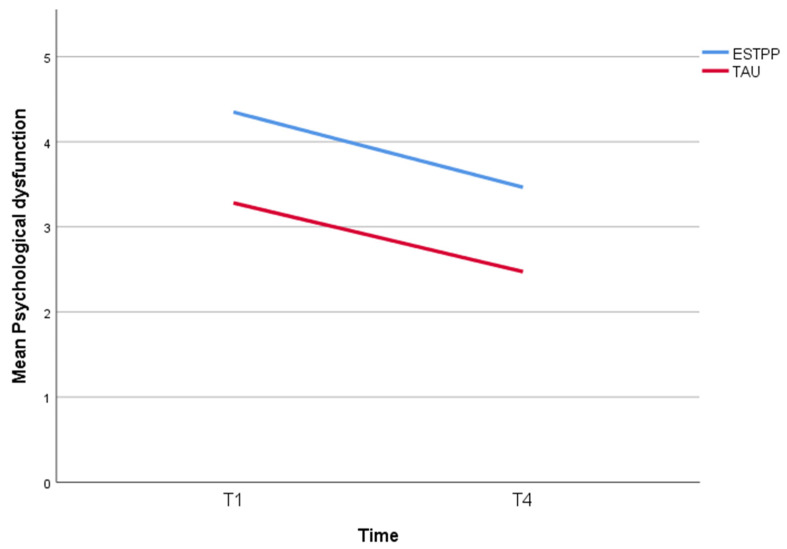
Effects of ESTPP and TAU on psychological dysfunction. Measurements were T1, at therapy start; T4, at 12 months after therapy start. Abbreviations: ESTPP, Equine-assisted Short-term Psychodynamic Psychotherapy; TAU, treatment-as-usual.

The mixed model revealed a significant decline in psychological dysfunction for both ESTPP and TAU over a 12-month period (resp. F(4) = 66.3, *p* < 0.001 and F(1) = 41.4, *p* < 0.001), with no significant difference between the two conditions (estimated effect, 1.2 [−0.7 to 0.3], *p* = 0.45) (see Table 4). Figure 1 depicts the slope of the decline in psychological dysfunction scores, which seems similar for both conditions.

### 3.2. Estimated Effects over Time for ESTPP

Table 5 shows the course of the outcome variables in the ESTPP condition over the time points. There were significant effects for time on psychological dysfunction, F(4, 406.98) = 66.34, *p* < 0.001; remoralization, F(4, 394.10) = 48.56, *p* < 0.001; and depression, F(4, 303.37) = 38.15, *p* < 0.001. Table 5 shows significant changes in outcomes when compared to T0 for all outcomes and time points but depression at T1. Additional analyses with T2 as a reference value, instead of T0, showed no significant effects at T4 for all the outcome variables, namely psychological dysfunction (0.07 [−0.26 to 0.39], *p* = 0.69), remoralization (−0.10 [−0.24 to.03], *p* = 0.14), and depression (1.13 [−1.13 to 4.12], *p* = 0.45).

Table 6 depicts that the courses of the outcomes for the preoccupied and fearful styles remain close to one another, whereas those for the dismissive style is closer to the secure style. Mixed models including an interaction term for the attachment styles with time points revealed non-significant interaction terms for all three outcome variables (psychological dysfunction F(12, 394.70) = 1.5, *p* = 0.12, remoralization F(12, 383) = 1.4, *p* = 0.18, depression F(12, 301) = 0.7, *p* = 0.79). Table 6 shows, compared to the secure style, a sharper fall in psychological dysfunction for the preoccupied style between T0 and T2 (−1.05 [−1.69 to −0.41], *p* < 0.001, *d* = 0.72) and a steeper growth of remoralization (0.43 [0.17 to 0.70], *p* = 0.02, *d* = 0.71). Furthermore, a sharper fall in psychological dysfunction scores between T0 and T4 can be seen for the fearful and dismissive styles, compared to the secure style.

### 3.3. Estimated Effects over Time of Internal Working Models of Self and Others (MoS, MoO) for ESTPP

For the Model of Self, although adding the interaction terms of MoS and time points was not significant for all three outcome variables, upon closer inspection a sharper fall in psychological dysfunction (−0.56 [−1.04 to −0.79], *p* = 0.02, *d* = 0.40.) and depression (−4.63 [−9.11 to −0.14], *p* = 0.04, *d* = 0.42) and a steeper growth in remoralization (0.24 [0.04 to 0.44], *p* = 0.02, *d* = 0.40) for negative MoS between T0 and T2 compared to positive MoS is discernable.

There seems to be a slightly steeper growth between T2 and T4 for psychological dysfunction and depression—and a slightly sharper fall for remoralization—for negative MoS between T2 and T4, compared to positive, although these effects are not significant (resp: −0.38 [−0.99 to 0.24], *p* = 0.23, *d* = 0.26; 0.55 [−5.03 to 6.13], *p* = 0.85, *d* = 0.05; 0.21 [−0.04 to 0.47], *p* = 0.10, *d* = 0.35) (see Table 6).

For the Model of Others, Table 6 depicts almost identical changes in psychological dysfunction, remoralization, and depression across all time points and for both positive and negative MoO. Although adding the interaction terms of MoO and the time points to the mixed models were not significant for all three outcome variables (psychological dysfunction F(4, 395) = 1.1, *p* = 0.37; remoralization F(4, 380) = 2.1, *p* = 0.08; depression F(4, 303) = 0.1, *p* = 0.98) Upon closer inspection, a less steep growth in remoralization between T0 and T2 (−0.27 [−0.47 to −0.07], *p* = 0.008, *d* = 0.45) for negative than for positive MoO is discernible. Then, a steeper fall in depression for negative MoO between T2 and T4 compared to T0 (0.45 [−5.43 to 6.32], *p* = 0.88, *d* = 0.04) is discernible (Table 6).

## 4. Discussion

The aim of this study was to gain insight into the role the attachment characteristics can play in treatment effects in adult patients with intrapsychic and interpersonal problems who underwent Equine-assisted Short-term Psychodynamic Psychotherapy (ESTPP) [41,57,70]. In the first part of the study, we compared the effects of ESTPP to treatment-as-usual regarding psychological dysfunction. We found that patients undergoing ESTPP seemed to benefit equally from TAU, with a moderate to large effect size. This finding of equal effectiveness, when two or more psychotherapies are compared, is consistent with the larger body of effectiveness research [5,92,93]. The results are similarly consistent with a study by Kovács et al. [41] on the effectiveness of ESTPP compared to TAU. They found equal effectiveness on different outcome variables, although self-esteem improvement during ESTPP was found to outperform TAU. Furthermore, with the present results demonstrating that the conditions were equally effective in ameliorating psychological dysfunction, this may suggest a greater efficiency of ESTPP. The latter entailed one intensive week of the therapy, followed by once-weekly sessions (or fewer), while TAU was delivered in three sessions per week over the same time-period of 12 months. The efficiency of both therapies should be explicitly examined in a cost-effectiveness study accounting for additional costs for ESTPP, such as travel expenses and horse-related overhead costs.

We then examined the course of ESTPP effects over the period of 1 year when controlled for attachment styles and, subsequently, for internal working models of self and others. We explored the outcome measures for psychological dysfunction, remoralization, and depression and the influence of attachment styles and Model of Self and Others. The distribution of attachment styles (i.e., secure, dismissive, fearful, or preoccupied) in our ESTPP condition proved comparable to that seen in clinical samples [94].

We found that, in addition to improvement in psychological dysfunction, patients saw significant improvements in remoralization and depression scores. These findings contribute to the body of research on EAP effectiveness in adult patients [70,95,96]. In addition, the prospect of undergoing ESTPP seemed to improve the course of pre-psychotherapy symptoms represented in the waiting time scores (T0–T1), which could indicate the building of epistemic trust in the offered intervention [96,97].

In line with prior studies [98,99,100], our results showed the highest psychological burden (high psychological dysfunction, low remoralization, and high depression) in patients with preoccupied and fearful attachment styles. Moreover, an association was found between type of attachment style and psychotherapy outcome. The results showed a larger decrease in psychological burden, reflected in the bigger changes in psychological dysfunction, remoralization, and depression for all insecure attachment styles, compared to the secure style (with a negligible to moderate clinical relevance over time). This proved especially true after the intensive psychotherapy module (T2) of ESTPP. This is in line with a study on effectiveness of intensive psychotherapy by Voorendonk et al. [101] in which an intensive 8-day treatment program ameliorated significant effects in PTSD-patients. However, our results also show that changes acquired in the intensive module were consolidated over a longer course of psychotherapy. This was particularly so for patients with a preoccupied attachment style. In contrast, patients with fearful or dismissive attachment styles gained improvement later over the course of the trajectory. Given that the dismissive-attached style predicts a greater rejection of psychotherapy and poorer psychotherapy outcome [102,103,104,105], these results for patients with a dismissive attachment style could be considered remarkable and therefore accounted for by the ESTPP. Further studies are needed to examine the added value of ESTTP for the dismissive patient specifically over non-EAP.

The improvements in outcome variables in relation to attachment characteristics in our study could be due to the attention given to interpersonal interactions and close relationships in ESTPP [57] and in AAP in general [35], in line with studies by Levy et al. [18] and Newman [106]. The latter found that patients who experience low pre-treatment attachment security may have better outcomes in psychotherapy that incorporates a focus on interpersonal interactions and close relationships. Moreover, Bernheim et al. [74] conclude that the patient can employ their capacity for synchrony in dyadic attachment situations to help resolve their interpersonal problems with sensitive and mutual interactions. In ESTPP, the patient is asked to form a mutual interaction with the horse based on synchronicity or mirroring on a non-verbal level [49,57,107]. This resembles the interpersonal interactions that, in our study, may have had a corrective influence on the impairments associated with attachment insecurity, in particular, for those with a dismissive attachment style.

### 4.1. Internal Working Models Self and Others in ESTPP

Our study found that the outcome variables for the preoccupied and fearful attachment styles were similar, whereas those of the dismissive attachment style proved similar to those of the secure attachment style. There was a similar finding for MoS. Patients with a negative MoS showed more psychological burden than those with a positive MoS throughout the trajectory. Interestingly, patients with a negative MoO followed a comparable patient journey as those with a positive MoO during ESTPP. For patients who have trouble in trusting others and can rely only on themselves, a horse could act as an opportunity to rely on the other. In line with Zilcha-Mano et al. [108], the presence of the horse in ESTPP (or another animal in AAP) may function as a “safe haven” in therapy for those who are so interpersonally traumatized that they are “beyond human attachment possibilities”, as may occur in patients with CPTSD, DESNOS, or DTD [16].

In line with the existing theory on the relationship between attachment styles and internal working models [32], patients with a negative internal MoS—corresponding to preoccupied or fearful style—reported more psychological burden than those with a positive MoS—corresponding to the dismissive style—who experienced less psychological burden. It seems that internal MoS differentiated more than internal MoO regarding the outcome variables. Therefore, MoS can be used to determine the focus of therapy by the clinician in ESTPP. It might be that MoO was less differentiated because more patients with a fearful attachment style (with higher reported psychological burden) and fewer patients with a dismissive style (with lower reported psychological burden) were represented in a negative MoO. In addition, negative MoO was less often observed in subjects characterized by preoccupied or secure attachment style. These findings call for more awareness among clinicians of MoS as Van Dijke et al. [25,27] suggested for patients with insecure attachment-based hyper- and de-activating self-regulation strategies rather than attachment style differentiation per se. It might be that the concept of internal working models aligns more easily than the attachment style differentiation to the perception of both clinician and patient, fostering the intersubjective therapeutic relationship. To our knowledge, no previous studies have differentiated for attachment styles and internal working models of self and others for EAP. Further studies are needed to examine the derivative of attachment styles from internal working models, whether these are competing models or supporting models for assessing focus in therapy.

Although not significant, a trend is observed at the end of the trajectory, with a fall in outcome variables for negative MoS and for the preoccupied and fearful style. This might suggest the importance of processing MoS in ESTPP as well, as Kovács et al. [41] also found for patients with personality problems and which is in line with a study by Dinger et al. [109]. Furthermore, these findings seem to suggest that, for preoccupied attached patients—negative MoS and positive MoO—a time-limited psychotherapy is preferable to encourage the patient to rely on themselves, whereas fearful attached patients—negative MoS and negative MoO—might need more time for self-esteem-enhancing activities. The internal working models [32] of the preoccupied and fearful attachment styles are characterized by feelings of being unsafe and unable to trust oneself. When the internal working MoS is processed in ESTPP, this could reduce experiences of shame, self-deprivation, fragmentation of self, or self-alienation. This should be examined in further studies.

The present findings show that, during ESTPP, patients with an internalized negative MoO follow a comparable patient journey as patients with an internalized positive MoO. Patients with a negative MoO reported significantly more remoralization (feelings of hope and trust) after the intensive module, and their depression seemed to improve comparable as well as that of those with a positive MoO over the course of ESTPP, although a steeper decline in depression for those with a negative MoO was observed at the end of the trajectory. For the patient with a negative MoO (i.e., difficulty trusting others), it seems that depression and remoralization are not different sides of the same coin: rather, gaining hope and confidence in the future seem more important than merely reducing depression.

A negative MoO is usually processed in psychotherapy through a positive therapeutic working alliance with the therapist in an experienced holding environment [110] and this seems to be provided during ESTPP. The results imply that a good-enough holding environment with a reliable therapeutic relationship can allow the therapy effects to process the internal working models of self. Further studies are needed to examine the relationship between the working alliance with the therapist and horse and ESTPP outcomes.

In short, preoccupied and fearful attachment styles show a similar course of therapy and represent most of the complaints of the study population. Patients with a dismissive and secure style also show a similar course, but with fewer complaints. During ESTPP, a different course is generated between negative Mos and positive MoS. Positive MoS generates a more favorable course of the different dependent variables (psychological dysfunction, remoralization, and depression). In addition, we see that negative MoS shows a trend in a relapse at the end of the trajectory (especially noticeable in the depression variable). Duration and intensity of therapy and a focus on MoS within therapy seem to be important elements. In MoO, we see that the course of negative MoO and positive MoO does not differ much from each other. It seems that MoO generates less differentiation on the various dependent variables over the course of therapy than MoS. So, at first, processing a negative concept of Self may seem more important than processing a negative concept of the Other in a therapy, or it could be that improving the Model of Self can have a beneficial effect on trust in the Other. From an attachment theoretical viewpoint [13,32], MoS and MoO are two mutually influencing models, which cannot be separated from each other, so processing MoS should affect MoO. The hypothesis arises that in an ESTPP, MoS could be more easily processed than in a non-animal assisted therapy, which should be further investigated.

### 4.2. Limitations

This investigation had several limitations. First, this study did not employ a randomized controlled design, which is still considered the gold standard for assessing effects of interventions. Due to the specific nature of animal-assisted therapies, it would be almost impossible to blind the patients or withhold information about the kind of psychotherapy from them. On the other hand, the advantage of a naturalistic design over RCT is the enhanced external validity. Second, the motivation for participating in the ESTPP intervention—which involved time abroad, in a remote wooded environment—could have influenced the scores, and this was potentially visible in the waiting list scores and could thus be considered as bias. However, no information was collected on patients’ specific preferences, interests, or fearfulness of horses, which could have influenced their participation in ESTPP. On the other hand, for the ESTPP patient, the prospect of working with animals in a therapeutic way could be interpreted as building epistemic trust in the intervention [96,97]. Third, no information on attachment style or remoralization and depression scores were gathered in the TAU group, which would have permitted a more thorough comparison between ESTPP and TAU. Fourth, only self-report instruments were used. No information was collected on how the therapists viewed the changes in their patients, and no information was gathered on therapy fidelity or adherence (psychotherapy integrity). However, one would expect a high quality of care because both interventions were executed and monitored by a specialized psychotherapy center and experienced professionals. Fifth, the division of the study sample into the different attachment styles and Model of Self and Others could have influenced the statistical power.

### 4.3. Future Research Aims

Besides the mentioned future research aims we suggest the following. The processing of the bodily-(emotional) level and of the muscle-motor activity in ESTPP [57], considered as parts of attachment and mentalization [111,112], is shown implicitly in this study. It would be interesting to explicitly study the degree of association between the bodily aspects of ESTPP or AAP and the outcome variables in future studies.

In addition, since attachment insecurity is intertwined with (interpersonal) trauma and personality problems, it would be useful to study the association between personality traits and the working alliance in ESTPP.

To establish the optimal psychotherapy options for patients with intertwined attachment and personality problems, there is a need to address the “what works for whom” question using a qualitative design in which the patients and therapists’ views on the degree of change are examined.

The established value of the intensive module in this study suggests a need to investigate trajectories with and without intensive modules.

In addition to further validate the added value of the horse in psychotherapy, further studies are valuable to determine the effectiveness of equines versus other animals, comparing interventions with other species.

## 5. Conclusions

Despite the limitations listed above, we can conclude that this study reveals a relevance for considering attachment characteristics, i.e., internal working models of self and others, in assessing focus in psychotherapy. Models of self and others could be more easily aligned with the experiences of both clinician and patient than the more general focus on symptom severity, DSM-5 disorders, or on attachment styles per se. Moreover, models of self and others may be used to predict the course of effects which is relevant for the question “what works for whom?”. In particular, duration and intensity of therapy and a focus on the Model of Self seem to be of relevance in ESTPP. In addition, ESTPP seems beneficial for patients with low pretreatment attachment security and particularly to provide for patients who need focus on the internal working Model of Self. ESTPP could be a preferred psychotherapy option for patients who lack (epistemic) trust in psychotherapy or are reluctant to change, such as those with a dismissive attachment style.

## Figures and Tables

**Table 1:** Measurement time points.

	T0 (2 Months before T1, Waiting List ESTPP)	T1 (Start Therapy)	T2 (after 1 Week Intensive Module ESTPP)	T3 (6 Months after Start ESTPP)	T4 (12 Months after Start)
**ESTPP**	BSI, RQ, BDI, ASQ	BSI, RQ, BDI	BSI, RQ, BDI	BSI, RQ, BDI	BSI, RQ, BDI
**TAU**		BSI			(BSI)

BSI = Brief Symptom Inventory; RQ = Remoralization Questionnaire; BDI = Becks Depression Inventory; ASQ = Attachment Style Questionnaire.

**Table 2:** Dummy variables for Model of Self (MoS) and Model of Others (MoO).

	MoS	MoO
**Secure**	1	1
**Fearful**	0	0
**Dismissive**	1	0
**Preoccupied**	0	1

Note: 1 indicates presence of positive MoS, MoO. 0 indicates presence of negative MoS, MoO.

**Table 3:** Descriptive of the study population at baseline (T0 for ESTPP and T1 for TAU).

	ESTPP		TAU	
	*M* (*SD*)	*N*	*M* (*SD*)	*N*
Age	37.6 (12.5)	193	30.5 (10.9)	107
Male		32 (16.6%)		29 (27.1%)
Psychological dysfunction	4.7 (1.5)	186	3.3 (1.2)	107
Remoralization	2.1 (.6)	180		
Depression	24.7 (11.1)	153		
**Attachment Style ESTPP**				
Secure		40 (20.2%)		
Preoccupied		82 (41.4%)		
Dismissive		37 (18.7%)		
Fearful		34 (17.2%)		

Abbreviations: ESTPP, Equine-assisted Short-term Psychodynamic Psychotherapy; TAU, Treatment-as-usual. Note: the number of participants in the ESTPP condition regards patients with a non-missing attachment style and a score on at least one of the outcome variables.

**Table 4:** Intervention effects on psychological dysfunction.

	ESTPP				TAU			
	*M* (*SD*)	*N*	*p*	*d*	*M* (*SD*)	*N*	*p*	*d*
***Measurement***								
T1	4.3 (1.4)	119			3.3 (1.2)	107		
T4	3.5 (1.6)	56			2.5 (1.2)	106		
**∆, *difference in scores*, *EE* (95%*CI*)**								
T4 vs. T1	−1.3 (−1.6 to −0.1)		<0.001	0.9	−0.8 (−1.1 to −0.5)		<0.001	0.7
ESTPP vs. TAU	−1.2 (−0.7 to 0.3)		0.45	0.8				

Measurements were T1, at therapy start; T4, at 12 months after therapy start. Abbreviations: ESTPP, Equine-assisted Short-term Psychodynamic Psychotherapy; TAU, treatment-as-usual; M, mean; SD, standard deviation; ∆, difference in scores, the reference values were T1, and ESTPP condition; EE, estimated effect; CI, confidence interval; *d*, standardized effect calculated as estimated effect divided by the standard deviation at T1.

**Table 5:** Results for mixed models with time points for outcome variables in ESTPP.

	Psychological Dysfunction			Remoralization			Depression		
	*M* (*SD*)	*N*	*p*	*M* (*SD*)	*N*	*p*	*M* (*SD*)	*N*	*p*
***Measurement***									
T0	4.7 (1.5)	186		2.1 (0.6)	180		24.7 (11.1)	153	
T1	4.3 (1.4)	119		2.3 (0.6)	115		22.2 (11.8)	86	
T2	3.3 (1.4)	103		2.7 (0.7)	104		15.3 (12.0)	74	
T3	3.2 (1.6)	115		2.6 (0.7)	113		15.5 (11.9)	91	
T4	3.5 (1.6)	56		2.6 (0.7)	56		16.3 (12.8)	48	
**∆, *scores compared to T*0, *EE* (95%*CI*)**									
T1	−0.2 (−0.5 to −0.001)		0.04	0.1(0.1 to 0.2)		0.003	−1.6 (−3.6 to 0.5)		0.14
T2	−1.4 (−1.6 to −1.1)		<0.001	0.6 (0.5 to 0.7)		<0.001	−10.5 (−12.7 to −8.2)		<0.001
T3	−1.5 (−1.8 to −1.3)		<0.001	0.5 (0.4 to 0.6)		<0.001	−10.3 (−12.4 to −8.2)		<0.001
T4	−1.3 (−1.6 to −1.0)		<0.001	0.5 (0.4 to 0.6)		<0.001	−9.3 (−12.1 to −6.6)		<0.001

Measurements were T0, at 2 months before starting the therapy, waiting list; T1, therapy start; T2, after 1 week of intensive ESTPP module); T3, 6 months after ESTPP start; T4, 12 months after ESTPP start. Abbreviations: M, mean; SD, standard deviation; **∆,** difference in scores compared to T0; EE, estimated effect; CI, confidence interval; ESTPP, Equine-assisted Short-term Psychodynamic Psychotherapy. Note: the number of participants in the ESTPP condition regards patients with a non-missing attachment style and a score of the outcome variable.

**Table 6:** Results for mixed models with attachment style and models of self–others in Equine-assisted Short-term Psychodynamic Psychotherapy (ESTPP). Differences in scores are presented as estimated effect (95% CI), *p*-value, and standardized effect-size *d*.

	Psychological Dysfunction			Re-Moralization			Depression		
	*EE* (95%*CI*)	*p*	*d*	*EE* (95%*CI*)	*p*	*d*	*EE* (95%*CI*)	*p*	*d*
∆ for pre-occupied									
T1	−0.4 (−1 to 0.2)	0.24	0.2	0.2 (−0.1 to 0.5)	0.12	0.3	−1.5 (−6.9 to 3.9)	0.98	0.1
T2	−1.1 (−1.7 to −0.4)	**0.001**	0.7	0.4 (0.2 to 0.7)	**0.002**	0.7	−4.3 (−6.4 to 7.6)	0.15	0.4
T3	−0.7 (−1.3 to 0)	**0.04**	0.5	0.1 (−0.1 to 0.4)	0.30	0.2	0.2 (−5.7 to 6.2)	0.94	0.02
T4	−0.7 (−1.5 to 0.1)	0.08	0.5	0.1 (−0.3 to 0.4)	0.74	0.01	−2.5 (−9.6 to 4.5)	0.48	0.2
∆ for fearful									
T1	−0.3 (−1 to 0.5)	0.48	0.2	0.1 (−0.2 to 0.4)	0.38	0.01	0 (−6.9 to 6.9)	0.10	0.002
T2	−0.6 (−1.3 to 0.2)	0.13	0.4	0 (−0.3 to 0.3)	0.10	0.002	−4.8 (−12.2 to 2.6)	0.21	0.4
T3	−0.6 (−1.3 to 0.1)	0.09	0.4	0 (−0.3 to 0.3)	0.84	0.1	−0.9 (−7.9 to 6.2)	0.81	0.1
T4	−1 (−1.9 to −0.1)	**0.04**	0.7	0.2 (−0.2 to 0.6)	0.43	0.3	0.6 (−7.9 to 9)	0.90	0.1
∆ for dismissive									
T1	0 (−0.7 to 0.7)	0.98	0.01	0.1 (−2.4 to 0.4)	0.70	0.2	0.1 (−6.4 to 6.6)	0.99	0.01
T2	−0.7 (−1.4 to 0.1)	0.07	0.5	0 (−0.3 to 0.3)	0.84	0.1	0.6 (−6.4 to 7.6)	0.86	0.1
T3	−0.7 (−1.5 to 0)	0.07	0.5	0.1 (−0.3 to 0.4)	0.74	0.1	0.1 (−7.2 to 7.3)	0.99	0.01
T4	−1 (−1.9 to −0.1)	**0.04**	0.7	−0.1 (−0.4 to 0.3)	0.74	0.2	−5.2 (−14.3 to 4)	0.27	0.5
∆ for MoS									
T1	−0.3 (−0.8 to 0.2)	0.20	0.2	0.2 (0 to 0.3)	0.12	0.3	−0.1 (−5.2 to 3.2)	0.64	0.1
T2	−0.6 (−1 to −0.8)	**0.02**	0.4	0.2 (0 to 0.4)	**0.02**	0.02	−4.6 (−9.1 to −0.1)	**0.04**	0.4
T3	−0.3 (−0.8 to 0.2)	0.19	0.2	0.2 (0 to 0.4)	0.06	0.3	0.1 (−4.3 to 4.5)	0.96	0.01
T4	−0.4 (−1 to 0.2)	0.23	0.3	0.2 (0 to 0.5)	0.10	0.4	0.6 (−5 to 6.1)	0.85	0.1
∆ for MoO									
T1	0.2 (−0.3 to 0.6)	0.45	0.1	−0.1 (−0.2 to 0.2)	0.63	0.1	1.2 (−3.2 to 5.7)	0.59	0.1
T2	0.1 (−0.4 to 0.6)	0.65	0.1	−0.3 (−0.5 to −0.1)	**0.008**	0.5	1 (−3.6 to 5.8)	0.64	0.1
T3	−0.13 (−0.6 to 0.2)	0.59	0.1	−0.1 (−0.3 to 0.1)	0.16	0.2	0 (−4.5 to 4.5)	10.0	0
T4	−0.47 (−1.1 to 0.2)	0.15	0.3	0 (−0.3 to 0.2)	0.91	0.02	0.5 (−5.4 to 6.3)	0.88	0.04

∆, difference in scores with secure attachment as a reference style, scores compared to T0, the first measurement at 2 months before starting the therapy, waiting list; Compared measurements were T1, therapy start; T2, after 1-week intensive module ESTPP); T3, 6 months after start ESTPP; T4, 12 months after start ESTPP. Abbreviations: ESTPP, MoS, Model of Self; MoO, Model of Others. Note: significant level < 0.05 in bold font.

## Data Availability

The dataset is available, upon reasonable request, and for research purposes only, by writing to the corresponding author.
